# Angiolymphoid hyperplasia with eosinophilia occurring in bilateral eyelids

**DOI:** 10.1186/1471-2415-13-38

**Published:** 2013-08-06

**Authors:** Shunichiro Ueda, Hiroshi Goto, Yoshihiko Usui, Takeshi Nagai, Toshitaka Nagao

**Affiliations:** 1 Department of Ophthalmology, Tokyo Medical University Hospital, 6-7-1 Nishihinjuku, Shinjuku-ku, Tokyo, 160-0023, Japan; 2 Department of Anatomic Pathology, Tokyo Medical University Hospital, Tokyo, Japan

**Keywords:** Angiolymphoid hyperplasia with eosinophilia, Kimura’s disease, Flow cytometry

## Abstract

**Background:**

Angiolymphoid hyperplasia with eosinophilia (ALHE) is an uncommon benign lesion, primarily occurring in the head and neck. ALHE arising from the ocular adnexa is rare, and the bilateral presentation is especially rare in the eyelids.

**Case presentation:**

A 64-year-old Japanese man presented with tearing. Multiple nodules, approximately 5 mm in size, were observed in bilateral upper and lower eyelids. Surgical excisions of the both eyelids masses were performed. Histopathological examination of the excised masses demonstrated proliferated blood vessels lined by plump endothelial cells together with a lymphoid and eosinopilic infiltrate, compatible with a diagnosis of ALHE. Flow cytometry studies showed that the mass consisted of mostly CD3-positive cells. During two-year follow-up, no recurrence of the mass was observed and the patient had no subjective symptom of tearing

**Conclusion:**

ALHE may occur in the bilateral eyelids. The cause of ALHE remains uncertain, but our results of flow cytemetry suggest that T cells are related to the pathogenesis of this disease.

## Background

Angiolymphoid hyperplasia with eosinophilia (ALHE) is an uncommon, poorly understood, benign slow-growing lesion [1]. The most common lesions seen in this entity are dermal or subcutaneous nodules of the face, scalp, neck and ears. Involvement of the ocular adnexa is rare compared with lesions of other sites [2,3]. The reported cases of ALHE occurring in the eyelid were unilateral [4,5].

We report a case of ALHE occurring in bilateral eyelids and discuss the clinical, histopathological and flow cytemetric features.

## Case presentation

**Figure 1:**
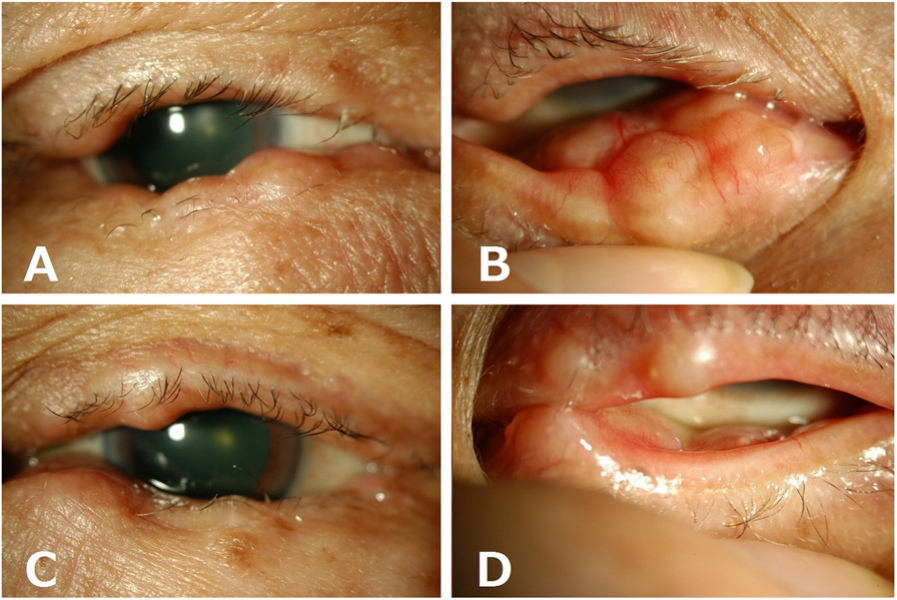
**Appearance of the eyelids at presentation. A**, **B**: right eyelid. **C**, **D**: left eyelid.

A 64-year-old Japanese man consulted a local clinic because of tearing. Multiple nodules each approximately 5 mm in size were observed in bilateral upper and lower eyelids. He was referred to our hospital for further examination and treatment. He had an unremarkable medical history and no history of trauma. The corrected visual acuity was 0.8 in each eye. Well defined hard lesions in both upper and lower eyelids were present, without inflammatory signs such as redness (Figure 1). Ophthalmoscopic findings were within normal limits except bilateral mild cataract changes. Laboratory investigations showed a white blood cell count of 10,400 per cm^3^ with 0.2% eosinophils. Serum concentration of immunoglobulin E was slightly elevated at 278.7 mg/dL, and immunoglobulin G4 was 59.3 mg/dL (normal range less than 135 mg/dL). Magnetic resonance imaging demonstrated bilateral multiple masses localized in the eyelids. The lesions were isointense to cerebral parenchyma on T1-weighted images, and hyperintense on T2-weighted images. After gadolinium infusion, the masses demonstrated moderate enhancement.

**Figure 2:**
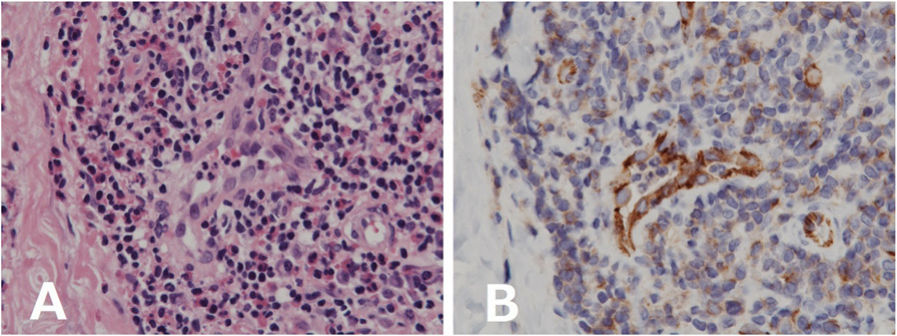
**Histopathological examinations of the biopsy. A**: Hematoxylin-eosin staining shows plump endothelial cells surrounded by collagenous stroma containing a lymphoid and eosinopilic infiltration; ×600. **B**: CD31 immunostaining highlights the prominent vascularity; ×600.

For diagnosis purpose, biopsy of the right upper eyelid mass was performed via the skin and conjunctiva. Since a pathological examination during surgery showed eosinophilic infiltration and an allergic disease was suspected, local injection of triamcinolone acetonide was performed at the end of procedures. Histopathological examination showed proliferated blood vessels lined by plump endothelial cells with an epithelioid appearance, surrounded by a collagenous stroma containing a lymphoid and eosinophilic infiltration, and no evidence of lymphoid follicles (Figure 2). These histopathological findings were compatible with a diagnosis of ALHE. In immunohistochemical studies, CD31 immunostaining highlighted the prominent vascularity and the plump endothelial cells. CD3 and CD20 immunostaining was diffusely positive in the infiltrate, indicating that most of the infiltrating cells were T cells. Flow cytometry studies showed that the mass was consisted of mainly CD3-positive cells, confirming the immunohistochemical findings. No evidence of IgH or T cell receptor (TCR) rearrangement was detected.

Subsequently the right eyelid mass was surgically excised. Residual lesions were removed piece by piece. At the end of surgery, triamcinolone acetonide was injected again into the eyelid. Although the patient refused additional surgery at first, one year after the initial operation, excision of left upper eyelid masses was performed in the same manner as the right eyelid mass. Histopathological and flow cytometric findings were the same as the right mass. During follow-up of two years, although some lesions remained unexcised, no recurrence of the excised masses was observed and the patient had no subjective symptom of tearing.

## Discussion

ALHE arising from the ocular adnexa is rare. ALHE occurring in eyelids which presented as bilateral multiple nodules has never been reported. The diagnosis of Kimura’s disease should be kept in mind in patients with a subcutaneous mass in the periocular region [6]. ALHE and Kimura’s disease are known to have similar clinical, laboratory and histopathological findings. Whether the two diseases are distinct or variations of the same disease have been discussed for many years. The main distinguishing feature between these two diseases is histopathological findings. Both diseases have an infiltrate of mostly lymphocytes and eosinophils, but ALHE is characterized by proliferating blood vessels lined by plump epithelioid endothelial cells, compared with the flat endothelial cells in Kimura’s disease. Moreover, elevations of blood eosinophils and serum immunoglobulin E in laboratory examinations characterize Kimura’s disease. Our case was diagnosed as ALHE instead of Kimura’s disease based on the differential features. Recently many studies have concluded that the two are distinct disorders [6].

The cause of ALHE remains uncertain. Our flow cytemetric study demonstrated that the mass was consisted of CD3-positive cells, but there was no TCR rearrangement. These findings suggest that the pathogenesis of ALHE might be a T cell reactive process. In some previous reports, a history of trauma or evidence of associated damage or rupture of blood vessels was present in many cases. Therefore, some authors considered ALHE as a reactive process. Moreover, the bilateral presentation in our case also suggests that there is a reactive process. On the other hand, others considered these lesions as neoplastic [7]. Previous papers have reported that these masses are consisted of mostly CD3-positive T cells [8] with monoclonality [9].

Complete surgical excision seems to be the most effective treatment [3]. However, facial ALHE is often impossible to remove completely. The recurrence rate after surgical excision is approximately 33% [10], and is especially higher after a partial removal [11]. Other treatments such as injection of corticosteroids and radiotherapy are generally not successful [10]. Our patient also resisted local steroid treatment although we tried several injections, but there was no recurrence in two years following surgical excision.

## Conclusion

ALHE may occur in the bilateral eyelids, albeit rarely. It is necessary to include ALHE in the differential diagnosis of an eyelid mass.

## Consent

Written informed consent was obtained from the patient for publication of this Case report and any accompanying images. A copy of the written consent is available for review by the Series Editor of this journal.

## Competing interests

The authors have declared that no competing interests exist.

## Authors’ contributions

SU: patient interaction and diagnosis, drafting of manuscript. HG: patient interaction and diagnosis, performed surgery. YU: patient interaction and diagnosis. TN: pathological diagnosis. TN: pathological diagnosis. All authors read and approved the final manuscript.

## Pre-publication history

The pre-publication history for this paper can be accessed here:

http://www.biomedcentral.com/1471-2415/13/38/prepub
